# Critical appraisal and meta-analysis of biological variation studies on glycosylated albumin, glucose and HbA_1c_

**DOI:** 10.1515/almed-2020-0029

**Published:** 2020-05-19

**Authors:** Carmen Ricós, Pilar Fernández-Calle, Elisabet Gonzalez-Lao, Margarida Simón, Jorge Díaz-Garzón, Beatriz Boned, Fernando Marqués-García, Joana Minchinela, Maria Carmen Perich, Xavier Tejedor-Ganduxé, Zoraida Corte, Aasne K. Aarsand, Berna Aslan, Anna Carobene, Abdurrahman Coskun, Sverre Sandberg

**Affiliations:** Spanish Society of Laboratory Medicine (SEQC^ML^), Analytical Quality Commission, Barcelona, Spain; Padilla, 323, Barcelona 08035, Spain; SEQC^ML^ , Analytical Quality Commission, Barcelona, Spain; EFLM, Task Group on Biological Variation Database; EFLM, Working Group on Biological Variation; and Hospital Universitario La Paz, Madrid, Spain; EFLM, Task Group on Biological Variation Database; and Quality Healthcare, Grupo ACMS, Madrid, Spain; EFLM, Task Group on Biological Variation Database; and Consortiumof Laboratory Intercomarcal Alt Penedès and Garraf l’Anoia, Vilafranca del Penedes, Spain; EFLM, TaskGroup on Biological Variation Database; and Hospital Royo Villanova, Zaragoza, Spain; EFLM, Task Group on Biological Variation Database; and Hospital Universitario de Salamanca, Salamanca, Spain; EFLM, TaskGroup on Biological VariationDatabase; and Hospital Germans Trias i Pujol, Badalona, Spain; EFLM, Task Group on Biological Variation Database; and Hospital Vall d’Hebron, Barcelona, Spain; EFLM, Task Group on Biological Variation Database; and Hospital Germans Trias i Pujol, Badalona, Spain; Hospital Universitario San Agustin, Aviles, Spain; EFLM, Task Group on Biological Variation Database; EFLM, Working Group on Biological Variation; Haukeland University Hospital, Bergen, Norway; Norwegian Quality Improvement of Laboratory Examinations, Haraldplass Deaconess Hospital, Bergen, Norway; EFLM, Task Group on Biological Variation Database; Institute for Quality Management in Healthcare of Canada, Toronto, Canada; EFLM, Task Group on Biological Variation Database; EFLM,Working Group on Biological Variation; and LaboratoryMedicine, Ospedale San Raffaele, Milan, Italy; EFLM, Task Group on Biological Variation Database; EFLM, Working Group on Biological Variation; and Acibadem Universitesi, Istanbul, Turkey; EFLM, Task Group on Biological Variation Database; EFLM,Working Group on Biological Variation; and Department of Global Public Health and Primary Care, University of Bergen, Bergen, Norway

**Keywords:** biological variation, biological variation critical appraisal checklist, biological variation database, diabetes mellitus

## Abstract

**Objectives:**

Numerous biological variation (BV) studies have been performed over the years, but the quality of these studies vary. The objectives of this study were to perform a systematic review and critical appraisal of BV studies on glycosylated albumin and to deliver updated BV estimates for glucose and HbA_1c_, including recently published high-quality studies such as the European Biological Variation study (EuBIVAS).

**Methods:**

Systematic literature searches were performed to identify BV studies. Nine publications not included in a previous review were identified; four for glycosylated albumin, three for glucose, and three for HbA_1c_. Relevant studies were appraised by the Biological Variation Data Critical Appraisal Checklist (BIVAC). Global BV estimates were derived by meta-analysis of BIVAC-compliant studies in healthy subjects with similar study design.

**Results:**

One study received BIVAC grade A, 2B, and 6C. In most cases, the C-grade was associated with deficiencies in statistical analysis. BV estimates for glycosylated albumin were: CV_I_=1.4% (1.2–2.1) and CV_G_=5.7% (4.7–10.6), whereas estimates for HbA_1c_, CV_I_=1.2% (0.3–2.5), CV_G_=5.4% (3.3–7.3), and glucose, CV_I_=5.0% (4.1–12.0), CV_G_=8.1% (2.7–10.8) did not differ from previously published global estimates.

**Conclusions:**

The critical appraisal and rating of BV studies according to their methodological quality, followed by a meta-analysis, generate robust, and reliable BV estimates. This study delivers updated and evidence-based BV estimates for glycosylated albumin, glucose and HbA_1c_.

## Introduction

Biological variation (BV) is defined as the random fluctuation of a measurand in a biological fluid to achieve a balance between turnover and the homeostatic setting point [1], [2], [3]. BV has two components: within-subject and between-subject variation, which may be expressed as variation coefficients (CV_I_ and CV_G_, respectively) [4]. In 1998, Fraser and Harris published a BV estimation model that has been widely used [1]. In 1999, the SEQC^ML^ Analytical Quality Commission [5, 6] compiled all publications containing BV estimates in a database that was updated every two years until 2014 [7]. The inclusion criteria employed have been extensively described by Perich et al. [8].

Despite the credibility and widespread use of the database created by Ricós et al., a number of authors have raised concerns about the accuracy and relevance of some of the BV estimates published in this database [9], [10], [11]. In response to this, the European Federation of Clinical Chemistry and Laboratory Medicine (EFLM) Working Group on Biological Variation (WG-BV) and the Task Group for the Biological Variation Database (TG-BVD) have undertaken several initiatives to improve the quality of available BV data, including developing a critical appraisal checklist to be applied to studies on BV, the *Biological Variation Data Critical Appraisal Checklist* (BIVAC) [12]. Applying this quality appraisal tool, the groups have been reviewing and critically appraising BV studies for a wide range of measurands and developed a meta-analysis approach to produce global BV estimates. The results of the critical reviews and meta-analysis derived global estimates for measurands associated with diabetes mellitus (DM) [13], lipids [14], and hematologic parameters [15] have recently been published.

Furthermore, the results of this systematic appraisal of BV data are being made publically available in the EFLM Biological Variation Database (EFLM BVD) [16]. This database was launched in the 23rd European Congress of Clinical Chemistry and Laboratory Medicine, *EuroMedlab* 2019 and is available at https://biologicalvariation.eu/ [16]. Presently, global BV estimates derived from meta-analysis have been published for more than 100 measurands and data are continuously being added. Thus, the new database provides quality-assured and updated data on the measurands most commonly tested in clinical laboratories for the management and monitoring of high-prevalence diseases.

One of the most prevalent diseases in the world is DM (8.5%) [17]. Accordingly, a wide range of international guidelines and recommendations have been published for the diagnosis and monitoring of glycemia. Glycosylated albumin has, for the last years, been attracting increasing attention of the scientific community [18], [19], [20] as an indicator of intermediate metabolism of glucose in settings that may interfere with the metabolism of hemoglobin.

Numerous papers on BV studies of diabetes-related measurands have been published over the last decade and our group has previously performed a systematic review and critical appraisal of BV studies for diabetes-related measurands, published in 2019 [13]. However, glycosylated albumin was not included in this systematic review as only one article had been published on this measurand to that date. Furthermore, high-quality studies such as the European Biological Variation Study (EuBIVAS) [21], a multicenter large-scale BV study was at the time of the previous review not yet published and thus not included. The objectives of this study were thus to perform a systematic review and critical appraisal for BV studies for glycosylated albumin and to deliver updated BV estimates for glucose and HbA_1c_, including recently published high-quality studies such as the EuBIVAS [21].

## Materials and methods

A systematic literature review and critical appraisal of BV studies were conducted for glycosylated albumin, glucose and HbA_1c_. A non time limited search for glycosylated albumin and furthermore, whereas for glucose and HbA_1c_ a complementary search was conducted only for the period from June 2018 and December 2019 (studies published after first review), using the same keywords and terms as in the previous review [13]. The search for studies related to glycosylated albumin was performed in the same manner, but without any time limit.

The BIVAC [12] is composed of 14 quality items and grades BV studies as A, B, C, and D in decreasing order of quality according to the level of compliance with each quality item. Quality item 1 assesses the scale of the measurand. Quality items 2–4 assess whether the characteristics of the subjects, samples, and measurement methods employed are described in sufficient detail, respectively. Quality items five to seven focus on pre-analytical and analytical conditions and whether steady state of participants has been assessed or data adequately transformed. Quality items 8–12 assess the measurement methods and statistical procedures used to derive CV_I_ and CV_G_ estimates. Quality items 13–14 refer to whether the number of results included in BV calculations and measurand concentrations are reported or not, respectively.

BV studies are graded as a function of the minimum score obtained for any of the quality items. A study showing full compliance with the 14 quality items is graded as “A”. If essential requirements defined for quality items 2, three and four are not complied with, the study will be rated as “D”.

Overall CV_I_ and CV_G_ estimates were derived from meta-analysis [22] of studies with similar study design performed in healthy adult individuals, by a weighted median approach. The BIVAC quality grades were used as weight, (A=4, B=2; C=1) together with inverse width of 95% confidence intervals (CI) of the CV_I_ and CV_G_ values obtained in each study. The 95% confidence intervals (95% CI) for the global estimates were calculated by the bias-corrected bootstrap method [23].

As described above, a meta-analysis was conducted to calculate the global BV estimates for each measurand. In the meta-analysis, all relevant studies fulfilling the inclusion criteria were included, i. e., both those identified in the previous review and those published after this review was finalized. The meta-analysis only included BV studies with a BIVAC grade A–C, based on a sample of more than three healthy adults (18–75 years of age) from whom more than three samples had been taken per subject at a once-a-month to twice-a-week sampling interval. For inclusion, studies were also required to describe BV results in detail and report estimates of analytical coefficients of variation (CV_A_). When stratified and overall BV data were provided in a publication (by age or sex, among other), only overall data were considered for the calculation of estimates to avoid duplicity of data.

## Results

The literature search identified six articles, dealing with healthy subjects. Of these only two were published after the BIVAC [12] (between March 2018 and December 2019) and four reported BV results for glycosylated albumin. Table 1 provides an overview of the appraised articles and for which measurands they have reported data.

**Table 1:** Papers revised in this study.

Year	Paper	Analyte	n
2019	Liang L, He H, Zeng Y, Zhang M, Wang X, Li X, Liang S et al. Evaluation of biological variation of glycated hemoglobin and glycated albumin in healthy Chinese subjects. J Clin Lab Anal 2019;33:322,715. https://doi.org/10.1002/jcla.2275.	Glycosylated albumin HbA_1C_	501
2018	Aarsand AK, Diaz-Garzón J, Fernandez-Calle P, Guerra E, Locatelli M, Bartlett WA et al. The EuBIVAS: Within- and between-subject biological variation data for electrolytes, lipids, urea, uric acid, total protein, total bilirubin, direct bilirubin, and glucose. Clin Chem 2018;64:1380-1393.	Glucose	335
2015	Parrinello CM, Lutsey PL, Couper D, Eckfeldt JH, Steffes MW, Caresh J et al. Total short-term variability in biomarkers of hyperglycemia in older adults. Clin Chem 2015;61:1540-1548	Glycosylated albumin	278
2013	Montagnana M, Paleari R, Danese E, Salvagno GL, Lippi G, Giuidi GC et al. Evaluation of biological variation of glycated albumin (GA) and fructosamine in healthy subjects. Clin Chim Acta 2013;423:1-4	Glycosylated albumin	273
2012	Xue L, Liang H, Jiang X. Circanual temperature-related variation in HbA_1c_ is unlikely to affect its use as a diagnostic test for type 2 Diabetes. Clin Lab 2012;58:481-488 [29].	Glucose	307
1993	Davie SJ, Whiting KL, Gould BJ. Biological variation in glycated proteins. Ann Clin Biochem 1993;30:260-264	Glycosylated albumin	31

Table 2 shows the number of articles which have been reviewed and their BIVAC grade. Glucose was the measurand with the highest number of publications. For the majority of studies, a BIVAC C grade was awarded (Table 2). Table 3 contains the overall CV_I_ and CV_G_ estimates derived by meta-analysis including all relevant studies.

**Table 2:** Number of articles reviewed and BIVAC grade for publications reporting BV estimates for DM related measurands.

		BIVAC grade			
Measurand	n	A	B	C	D
Glycosylated albumin	4	0	1	3	0
HbA_1c_	3	0	1	1	1
Glucose	7	1	0	6	0
Previous systematic revision [13]					
Glycosylated albumin	NA	NA	NA	NA	NA
HbA_1c_	17	1	2	10	4
Glucose	23	2	1	20	0

n, number of articles reviewed; NA, not assessed.

**Table 3:** Overall estimates for BV components.

Measurand	CV_I_% (95% CI)	CV_G_% (95% CI)
Glycosylated albumin	1.4 (1.2–2.1)	5.7 (4.7–10.6)
HbA_1c_	1.2 (0.3–2.5)	5.4 (3.3–7.3)
Glucose	5.0 (4.1–12.0)	8.1 (2.7–10.8)

CV_I_, within-subject biological variation.

CV_I_, between-subject biological variation.

95% CI, 95% confidence interval.

**Figure 1:**
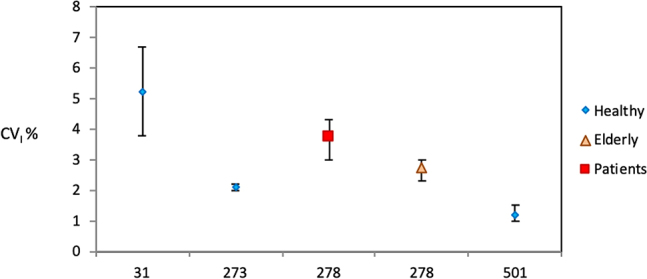
Glycosylated albumin. CV_I_ and their confidence intervals for the four papers included in this review. X axis: paper ID number, according to Table 1.

Figure 1 shows the reported CV_I_ estimates of glycosylated albumin, a measurand that was not included in the previous review [13]. Notably_,_ CV_G_ estimates were only reported in three papers, with the most recent study reporting lower values than previous studies, as follows: CV_G_=4.7 vs. 10.3% and 10.7%.

## Discussion

The clinical relevance of the measurands used for the diagnosis and monitoring of DM makes it necessary that BV estimates for these measurands are robust and reliable. BV data are also used to produce analytical imprecision and bias specifications, which are essential for an optimal laboratory performance. In addition, reference change values are calculated based on BV, which enables an appropriate interpretation of serial results [2, 3, 24].

Although glucose and HbA_1c_ traditionally have been used for the diagnosis and monitoring of glycemia, some authors advocate the use of glycosylated albumin for the diagnosis, prevention, and monitoring of diabetes in diabetic patients in clinical settings that may interfere with hemoglobin metabolism [18], [19], [20]. Hence, the relevance of providing robust BV estimates to laboratories using this monitoring strategy.

As in the previous systematic review, appraisal by the BIVAC demonstrates that the majority of studies published for diabetes-related measurands have similar methodological weaknesses [13]. Summarizing all the 75 studies (first and present reviews) most were graded as “C” regardless of the measurand addressed for the following reasons: outliers were not identified and removed (quality item 8) in 86%; homogeneity of variance had not been assessed in 64% (item 10). In 50%, CI for BV estimates were not reported, could not be calculated, or the number of results excluded were not documented (quality items 12 and 13, respectively); CV_A_ estimates were calculated based on internal quality control instead of replicate analyses of samples in 60 and 30% of the papers included in the first and the second review, respectively (quality item 6); steady state of the patient during the study period was not assessed in 20% (quality item 7).

The methodological flaws of studies may affect the reliability of the resulting estimates. Failure to identify outliers (quality item eight of BIVAC) and assess the homogeneity of variance (quality item 10), may cause CV_I_ and CV_G_ estimates not to be generalizable or reliable.

The lack of data on the total number of results used to calculate BV estimates (quality item 13) in many studies makes it difficult to assess group homogeneity and the generalizability and reliability of the estimates.

A BIVAC grade A requires that BV studies estimate CV_A_ by replicate analysis of study samples (quality item 6) [1, 12].

Another relevant aspect is assessing the steady state of subjects during the study period (quality item 7), since variations in concentrations may result in inaccurate CV_I_ and CV_G_ estimates.

One of the strengths of BIVAC is that the meta-analysis model employed to produce a final CV_I_ and CV_G_ estimate gives priority and more weight to BV data derived from BIVAC compliant studies.

For glucose, two new articles which fulfilled the inclusion criteria were identified in this review. These were included in the meta-analysis to provide the updated estimates. However, the updated estimates did not differ from those published in the previous review (Table 2).

For HbA_1c_, the CV_I_ and CV_G_ estimates were also similar to those reported in the previous review [13], with a narrower CI that may be explained by the inclusion of a high-quality study graded as B [25] (number 501 in Table 1).

A considerable dispersion of the CV_I_ values reported for glycosylated albumin in the four studies conducted in healthy adults was observed, with the two most recent studies (numbers 273 [26] and 501 [25], from 2013 to 2019, respectively) reporting lower CV_I_ values (Figure 1). Three articles were included in the meta-analysis, whereas one ([27] number 31) was excluded as CV_A_ values were not reported and CIs could not be calculated. Significantly higher estimates were documented in a study in subjects older than 75 and in a study in diabetic subjects [28] (number 278 in Figure 1).

The high CV_I_ obtained in the earliest paper [27] (number 31 in Figure 1) from 1993) can be explained by the use of an outdated analytical method (affinity chromatography), as compared to the other articles, where more specific analysis (automated enzymatic), was used. A study [25] involving Chinese population reports a lower CV_I_ estimate, which might be explained by ethnicity-based differences.

These estimates CV_I_=1.4% (1.2–2.1), CV_G_=5.7% (4.7–10.6) differ remarkably from those contained in the 2014 database [7] (CV_I_=5.2% and CV_G_=10%), as only data from the study based on affinity chromatography were included in the former database. Further high-quality studies are required to produce reliable CV_I_ and CV_G_ estimates for glycated albumin.

The BIVAC was published in March 2018. Only two studies were identified in this review that was published after the BIVAC became publically available. These two papers, one study on glucose and one glycosylated albumin and HbA_1C_, were graded as A [21] (number 335 in Table 1) and B [25] (number 501 in Table 1), respectively, which may indicate that the authors followed the BIVAC to assist in their BV study design and analysis. However, one of these studies is the EuBIVAS, which is published by the WG-BV, who have followed this design consistently also prior to the BIVAC. Therefore, though one of the aims of the BIVAC is to improve in the quality in future studies; it is too early to draw any conclusions on this].

## Conclusions

The application of the BIVAC to evaluate the quality of BV studies is a standardized method that aids in the production of robust and reliable BV estimates. In our study, we have delivered global BV estimates for glycated albumin based on critical appraisal and meta-analysis of relevant studies. The inclusion of recently published studies for glucose and HbA_1c_ did not significantly impact the point estimate delivered by our previous review. However, the future inclusion of a higher proportion of high-quality studies may have a greater impact and is likely to progressively decrease the width of the CI.

The impact of BIVAC is not restricted to the appraisal of publications, but it also provides an international standard for the design, performance, and publication of BV studies, and provides standard guidelines for the design of new BV studies.

The scarcity of studies receiving the highest BIVAC grade demonstrates the need for more fully BIVAC-compliant studies to provide robust BV estimates. The use of the BIVAC for the design and development of studies may be instrumental to improve the quality and reliability of BV estimates in the future.
